# The Potential Role of Deubiquitinating Enzymes (DUBs) in Male Fertility

**DOI:** 10.3390/biom16020299

**Published:** 2026-02-13

**Authors:** Jung Min Kim

**Affiliations:** Department of Pharmacology, Chonnam National University Medical School, Hwasungun 58128, Republic of Korea; jungminkim@jnu.ac.kr

**Keywords:** ubiquitination, deubiquitinating enzymes (DUBs), spermatogenesis, DNA repair, genome instability, male infertility

## Abstract

Ubiquitination dynamically regulates critical cellular processes, including cell cycle progression, apoptosis, DNA repair, and chromatin remodeling. Deubiquitinating enzymes (DUBs) negatively regulate protein ubiquitination and are equally important for protein regulation in diverse biological processes. Spermatogenesis is a highly regulated process involving spermatogonia self-renewal and differentiation, ensuring continuous sperm production. Using a loss-of-function mouse model, several DUBs have been shown to be involved in spermatogenesis. In addition, specific genetic variants in the *DUB* genes have been associated with spermatogenic failure and male infertility. These studies provide strong evidence that DUBs are necessary for normal spermatogenesis and may influence male fertility. However, the exact mechanism by which these DUBs regulate spermatogenesis is still under investigation. The aim of this review is to highlight recent advances in the regulatory roles of DUBs in mammalian spermatogenesis and provide insight into the molecular mechanisms underlying their potential actions. An in-depth understanding of DUB-mediated regulation of spermatogenesis will provide a scientific rationale for the discovery and development of novel DUB-targeted therapeutic strategies for male infertility.

## 1. Introduction

### 1.1. Spermatogenesis

**Figure 1:**
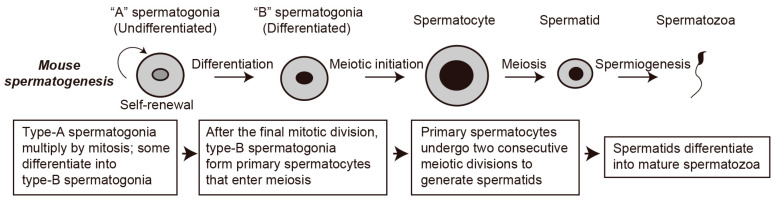
Mammalian spermatogenesis. Proliferative type A spermatogonia (SSCs) undergo a series of mitotic divisions. One of the daughter cells renews the stock of type A spermatogonia (undifferentiated spermatogonia), while the other becomes type B spermatogonia (differentiating spermatogonia). Type B spermatogonia give rise to primary spermatocytes, which undergo two meiotic divisions to produce haploid spermatids that undergo spermiogenesis to produce terminally differentiated spermatozoa.

Mammalian spermatogenesis has three key phases of germ cell development, namely, the proliferation of spermatogonia, meiosis in spermatocytes, and post-meiotic differentiation in spermatids [1,2,3]. Spermatogonia are diploid germline stem cells located in the seminiferous tubules of the testes. They undergo mitosis to produce two types of daughter cells. Type A spermatogonia (spermatogonial stem cells, SSCs) remain near the basement membrane of the tubule to maintain the supply of germline stem cells [2,4,5]. After a series of mitotic divisions, type B spermatogonia grow and differentiate into primary spermatocytes [2,6]. Diploid primary spermatocytes subsequently undergo two meiotic divisions and result in the production of haploid round spermatids. They then enter the process of spermiogenesis during which profound morphological and biochemical restructuring occurs, such as the formation of the acrosome and flagellum, giving rise to mature spermatozoa [7] (Figure 1).

### 1.2. The Ubiquitin–Proteasome System (UPS)

**Figure 2:**
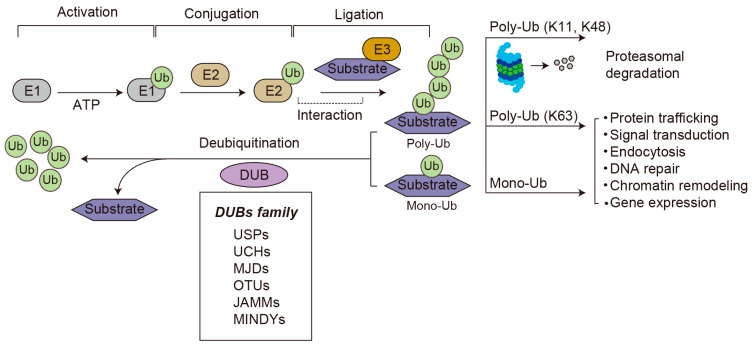
An overview of the ubiquitin–proteasome system (UPS). Ubiquitin is activated with E1 (ubiquitin-activating enzyme) in an ATP-dependent manner, transferred to E2 (ubiquitin-conjugating enzyme), and then transferred to the substrate through E3 (ubiquitin ligase) recognition, forming a mono- or polyubiquitinated protein. K48 or K11 polyubiquitin chains lead to 26S proteasome-mediated degradation. Monoubiquitination or K63 polyubiquitin chains are non-proteolytic ubiquitination signals and participate in many biological processes. Deubiquitination is mediated by deubiquitinating enzymes (DUBs) that remove ubiquitin from substrates, allowing for ubiquitin recycling. The DUB family consists of six subgroups, USPs, UCHs, MJDs, OTUs, JAMMs, and MINDYs, classified based on the characteristics of their conserved domains.

Ubiquitination is an important protein post-translational modification regulating many cellular processes in eukaryotes. This modification can mark proteins for degradation by the proteasome, or it can act as a signal for non-proteolytic functions of proteins [8,9,10]. Ubiquitination occurs through covalent attachment of a ubiquitin molecule to substrate lysine residue mediated by E1-activating, E2-conjugating, and E3 ubiquitin ligase enzymes [11] (Figure 2).

The proteins can be conjugated with a single ubiquitin molecule (monoubiquitin) or chains of ubiquitin (polyubiquitination). Ubiquitin contains seven lysine residues (K6, K11, K27, K29, K33, K48, and K63) that can undergo distinct polyubiquitination chains. The polyubiquitinated chain using K48 is typically used as means of protein expression control and directs the protein to the 26S proteasome for degradation [12]. It has recently been demonstrated that linkages at other lysine residues such as K11, K29, and K33 also serve as signals for proteasomal targeting but are less common [13,14,15]. Compared with this, monoubiquitination or K63-linked polyubiquitination exerts non-proteolytic functions, such as protein trafficking, endocytosis, DNA repair, transcriptional regulation, and signal transduction.

Deubiquitinating enzymes (DUBs) reverse the process of ubiquitination and play critical roles in various cellular processes [8,16,17,18]. To date, more than one hundred human DUBs have been identified with varying substrate specificity, subcellular localization, and protein–protein interactions [19]. DUBs are classified into six families according to their domain structure: ubiquitin-specific proteases (USPs), which comprise the largest and most diverse subfamily, ubiquitin carboxy-terminal hydrolases (UCHs), ovarian tumor proteases (OTUs), Machado–Joseph disease protein domain proteases (MJDs), JAMM/MPN domain-associated metallopeptidases (JAMMs), and monocyte chemotactic protein-induced proteases (MINDYs) [16,20]. With the exception of JAMMs, which belong to the Zn^2+^-dependent metalloproteases, the others use the classical cysteine protease catalytic triad (Cysteine–Histidine–Asparagine/Aspartate) in the active site. Similar to E3 ubiquitin ligases, DUBs have specificity toward different ubiquitin linkage types and the substrate protein. Since DUBs function to antagonize the ubiquitination of substrates carried out by E3 ligase, the levels of ubiquitination are dynamically regulated by the actions of these two opposing enzymes, which are regulated at expression, subcellular location, or activity levels through mechanisms by protein-protein interactions and post-translational modifications.

## 2. Maintenance of Genome Integrity During Spermatogenesis

**Figure 3:**
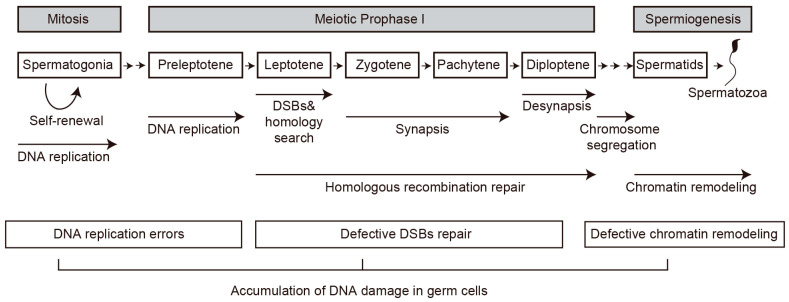
A schematic representation of the stages of progression, the events, and the potential reasons of DNA damage occurring during mammalian spermatogenesis. The main events in each cellular phase (spermatogonia, preleptotene, leptotene, zygotene, pachytene, diplotene, spermatids) are marked with arrows.

Spermatogenesis is divided into three phases: the mitotic phase of spermatogonia, the meiotic phase of spermatocytes, and the post-meiotic phase of spermatids (spermiogenesis) (Figure 3). The rapid mitotic division of spermatogonia has an increased risk of spontaneous DNA damage, primarily through DNA replication errors [21,22,23,24,25,26]. While these cells have various DNA repair mechanisms to maintain genome stability, unrepaired DNA damage can lead to the accumulation of mutations. Meiotic DNA double-stand breaks (DSBs) are generated early in meiotic prophase I and initiate homologous recombination (HR), producing genetic diversity and ensuring accurate transmission of genetic information to the next generation [27,28,29,30]. Defects in DSB repair can cause abnormal recombination and meiotic arrest in primary spermatocytes, leading to massive germ cell death [30,31,32]. During spermiogenesis, round spermatids undergo dramatic chromatin remodeling with histone replacement by protamine and subsequently differentiate into mature spermatozoa [33]. Defective histone-to-protamine replacement can lead to incomplete chromatin condensation and DNA damage. As sperm mature, DNA damage can be accumulated by various risk factors including oxidative stress, chromatin remodeling errors, and unrepaired DNA damage carried over from earlier stages, leading to increased sperm DNA fragmentation and consequently defective sperm production and infertility.

The five main DNA repair mechanisms involved in the maintenance of genome integrity include base excision repair (BER), nucleotide excision repair (NER), HR repair, non-homologous end-joining (NHEJ) repair, and mismatch repair (MMR) [21,22,23,24,34]. An increasing number of studies have demonstrated that genetic variants in DNA repair-related genes are associated with male infertility [30,35,36,37,38]. In particular, MMR and HR repair have been most closely linked to male infertility [32,39]. For example, genetic variants in MSH4/MSH5 (MMR genes) and BRCA1/BRCA2/NBS1 (HR repair genes) have been shown to be associated with spermatogenetic failure in men and mice [32,39,40,41,42,43,44]. Many DUBs play a crucial role in regulating DNA damage repair and ensuring genomic stability [45,46]. By directly deubiquitinating DNA repair proteins, DUBs regulate their stability and repair activity, thereby promoting efficient DNA damage repair. In addition, DUBs can also regulate the accessibility of DNA repair proteins to sites of DNA damage by affecting the ubiquitination status of histones [45,47,48,49]. Recently, genetic variants in certain DUBs involved in DNA repair were found in men with non-obstructive azoospermia (complete absence of sperm in semen) [50]; however, it is currently not clear whether these mutations are directly associated with spermatogenic failure or whether they affect the DNA repair activity of DUBs. Further investigation is needed to elucidate the causal relationships between these genetic variants and male infertility.

## 3. Specific E3 Ubiquitin Ligases and Their Influences on Spermatogenesis

Spermatogenesis is tightly regulated by ubiquitination, which ensures that specific proteins are active only during the correct stages of germ cell development [51,52]. An increasing number of studies have revealed that the ubiquitin–proteasome system (UPS) is required for normal spermatogenesis, and impairment of the UPS causes severe defects in germ cell development, increased DNA damage, and male infertility [53,54,55,56]. During spermatogenesis in mammals, more than 70 distinct E3 ubiquitin ligases are expressed and execute diverse functions at different stages of spermatogenesis, including spermatogonia differentiation, meiosis, spermiogenesis, and spermiation (sperm release) [52,57,58,59]. 

HUWE1 (HECT, UBA, and WWE domain-containing protein 1) is a large HECT domain E3 ligase that controls the balance of SSC self-renewal and differentiation by regulating DNA damage response [53,60]. *Huwe1*-deficient mice show depletion of spermatogonia, spermatocytes, and extensive loss of spermatids, resulting in male infertility [53]. Additionally, HUWE1 is involved in Sertoli cell development by mediating the ubiquitination and degradation of WT1 (Wilms Tumor 1), a critical transcription factor specifically expressed in Sertoli cells [61]. 

CUL4A (Cullin-4A) and CUL4B (Cullin-4B) are ubiquitin ligase components of multimeric complexes involved in cell cycle progression, DNA repair, and other processes. CUL4A and CUL4B are essential, non-redundant E3 ubiquitin ligases that regulate distinct stages of spermatogenesis [62,63,64]. CUL4A is highly expressed in primary spermatocytes, while CUL4B is expressed in spermatogonia, Sertoli cells, and post-meiotic spermatids. CUL4A is critical for meiotic progression by maintaining genome stability. *Cul4a*-deficient mice exhibit extensive germ cell apoptosis due to the persistent DNA damage in pachytene spermatocytes, suggesting a deficiency in homologous recombination repair [62,63]. Unlike CUL4A, CUL4B is required for spermatid development (spermiogenesis), with its loss causing impaired spermatid maturation, reduced motility, and a low sperm count [64]. 

RNF126 (Ring finger protein 126) and UBR2 (Ubiquitin protein ligase E3 Component N-Recognin 2) are involved in homologous recombination repair and the maintenance of genomic integrity during meiosis I [65,66]. *Rnf126*-deficient mice exhibit reduced fertility characterized by spermatogenic arrest in meiotic prophase I [65]. Moreover, *RNF126* gene variants (SNPs) are associated with non-obstructive azoospermia and oligozoospermia [65], highlighting the potential of RNF126 as a diagnostic marker for male infertility. *Ubr2*-deficient mice are infertile due to the deficiency in homologous chromosome pairing (synapsis), resulting in increased apoptosis in pachytene spermatocytes [66]. In particular, UBR2 mediates histone H2A ubiquitination (H2AK119ub), which induces transcriptional silencing of the sex chromosomes (X, Y) during meiosis I [67]. 

RNF8 (Ring finger protein 8) plays a critical role in spermatogenesis by ubiquitinating histones, primarily H2A and H2AX. RNF8-dependent histone ubiquitination is essential for chromatin remodeling, driven by histone-to-protamine exchange during spermatid maturation, and its deficiency leads to abnormal spermatozoa, resulting in male infertility [68,69].

PHF7 is critical for histone-to-protamine replacement during spermiogenesis by regulating histone ubiquitination (H2A and H3) and BRDT (Bromodomian testis associated) stability [70,71]. A loss of *Phf7* leads to spermatogenic failure, characterized by impaired chromatin condensation in spermatids, resulting in male infertility. 

RNF133 is an endoplasmic reticulum (ER)-anchored E3 ligase that plays a critical role in sperm development and fertility [72,73]. *Rnf133*-deficient mice display severe subfertility, characterized by abnormal morphology and reduced motility of sperm. RNF133 may play a role in ER-associated degradation (ERAD) by promoting the ubiquitination of target proteins to ensure male fertility. 

ASB17 (Ankyrin repeat and SOCS box-containing 17) is a testis-specific E3 ligase that regulates the final stage of sperm release (spermiation) from the seminiferous tubules by degrading actin-binding protein Espin (ESPN) at the ectoplasmic specialization (ES) junction [74]. *Asb17*-deficient mice are fertile with normal testicular development, but they exhibit oligospermia due to failed sperm release and reduced germ cell apoptosis.

## 4. The Role of DUBs in Spermatogenesis

**Figure 4:**
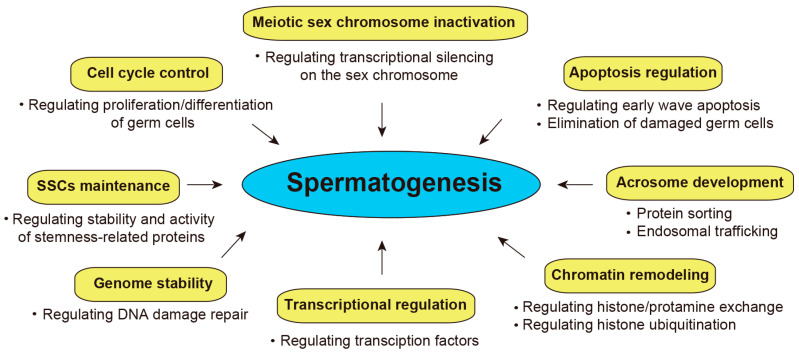
Schematic representation of critical role of DUBs involved in mammalian spermatogenesis (for details, see Section 4).

DUBs provide a critical regulatory layer by counteracting E3 ubiquitin ligase, potentially ensuring the precise control of protein modifications necessary for spermatogenesis. DUBs can play a critical role in different stages of spermatogenesis through diverse mechanisms (Figure 4): (i) SSC self-renewal: Spermatogonial stem cells (SSCs) undergo either self-renewal or differentiation to support spermatogenesis [75]. Recent studies have shown that certain DUBs, such as USP3, USP7, USP21, and USP22, are involved in stem cell maintenance by controlling the stability of core pluripotency factors such as Oct4, Sox2, and Nanog [76,77,78,79,80]. Although their specific roles in SSC maintenance require further investigation, DUBs may play a role in determining the fate of SSCs by regulating the stability of stemness-related proteins [81,82]. (ii) Cell cycle regulation: DUBs can regulate the cell cycle by deubiquitinating cell cycle regulators, which influences the proliferation and differentiation of undifferentiated spermatogonia [2,75]. (iii) Genome maintenance: DNA damage can occur during spermatogenesis. DUBs involved in DNA repair pathways play an important role in maintaining the genome integrity of germ cells [45,46]. (iv) Apoptosis regulation: During the first round of spermatogenesis, excess pre-meiotic spermatogonia are intentionally removed by an early wave of apoptosis [83]. Additionally, germ cell apoptosis is also required for the removal of damaged germ cells during both development and adult life, thereby maintaining genomic integrity. DUBs involved in the control of apoptosis play a role in maintaining testicular homeostasis. (v) Meiotic chromosome silencing: meiotic sex chromosome inactivation (MSCI) is essential for transcriptional silencing of sex chromosomes (XY body) during meiosis [84]. DUBs can influence MSCI by regulating H2A monoubiquitination, thus ensuring proper meiotic progression [85,86,87,88]. (vi) Transcriptional regulation: DUBs can influence the activity of transcription factors either directly or indirectly via deubiquitination, and are thus involved in transcriptional regulation [47,88,89]. (vii) Chromatin remodeling: During spermiogenesis, extensive chromatin remodeling occurs in spermatids, which involves histone-to-protamine exchange for chromatin condensation [33]. By regulating histone ubiquitination (H2A and H2B), DUBs may contribute to proper chromatin remodeling, which is crucial for sperm maturation and function [33,89,90]. (viii) Acrosome development: The acrosome is a specialized organelle that covers the anterior part of the sperm nucleus and is essential for successful fertilization. Since monoubiquitination serves as a signal for protein sorting, trafficking, and localization during acrosome assembly, DUBs are therefore involved in this process by regulating the balance of ubiquitination and deubiquitination of acrosomal proteins. The failure of these ubiquitination-related trafficking pathways can lead to defects in acrosome formation and result in defective sperm function.

## 5. DUBs Potentially Associated with Male Infertility

This section will highlight the functional roles and molecular mechanisms of DUBs that are (potentially) involved in mammalian spermatogenesis (Table 1).

### 5.1. USP1

USP1 (Ubiquitin-specific protease 1) is a well-characterized DUB that plays an important role in the cellular response to DNA damage [48,91,92]. *Usp1*-deficient mice are infertile, characterized by the absence of germ cells, with only Sertoli cells remaining in the seminiferous tubules [93]. UAF1 is a cofactor that stimulates the activity of several DUBs, including USP1, USP12, and USP46 [94,95]. Similarly, germ cell-specific *Uaf1*-deficient mice are infertile, characterized by a reduced number of epididymal spermatozoa and defects in spermiogenesis [96], suggesting that the USP1/UAF1 complex is essential for normal spermatogenesis. Although the exact mechanism by which USP1 deficiency leads to complete infertility is not fully understood, the most likely mechanism may be the role of USP1 in DNA damage repair. USP1 deubiquitinates the FANCD2/FANCI complex, a key step in the Fanconi Anemia (FA) pathway that repairs DNA interstrand crosslinks (ICLs) [48,92]. As expected, USP1 deficiency impairs the FA pathway, leading to defective HR repair and increased genomic instability in mice [93]. Similarly, FA mouse models consistently exhibit male infertility [97,98], a characteristic of FA patients. Moreover, recent studies have shown that genetic variants in FA genes have been linked to male infertility including non-obstructive azoospermia [99,100]. Defects in DNA repair by mutations in FA genes may cause spermatogenic arrest, ultimately resulting in male infertility. USP1 is also involved in Proliferating Cell Nuclear Antigen (PCNA)-mediated translesion synthesis (TLS) [91]. By deubiquitinating PCNA, USP1 negatively regulates error-prone TLS activity and therefore can reduce the risk of genome instability in germ cells [91,101]. However, DNA repair defects cannot solely account for the complete loss of germ cells in *Usp1*-deficient mice; these phenotypes might be attributed to a combination of multiple mechanisms, including cell cycle regulation, transcription regulation, and chromosome remodeling. USP1 deubiquitinates and stabilizes inhibitor of differentiation (ID) proteins, including ID1, ID2, and ID3, which belong to the basic helix–loop–helix family of transcription factors [102]. By preventing degradation of ID proteins, USP1 may contribute to self-renewal of SSCs. Importantly, a recent study has identified rare genetic variants in the USP1 gene in male patients with non-obstructive azoospermia [50], but how these mutations affect USP1 function remains largely unknown. Moreover, a reduced level of USP1 expression was found in an azoospermic male with a reciprocal translocation of 46,X,t(Y;1)(p11.3;p31) [103]. Collectively, these studies suggest that USP1 is crucial for normal spermatogenesis, and further investigation is needed to fully understand the mechanisms by which USP1 influences spermatogenesis.

### 5.2. USP2

USP2 is highly expressed in elongated spermatids. In *Usp2*-deficient mice, the overall process of spermatogenesis is morphologically normal, but there are some abnormalities during the final stages of sperm maturation [104]. Sperm from *Usp2*-deficient mice have significantly reduced motility, which is particularly sensitive to environmental changes, suggesting that the sperm’s ability to sustain motility outside of the body is compromised. Moreover, the fertilization capacity of sperm from *Usp2*-deficient mice is severely impaired, particularly in the process of penetrating the outer layer of the egg (zona pellucida), suggesting that USP2 is essential for the stabilization of proteins within the spermatid, ensuring proper sperm structure and function during late development. Although the molecular mechanism or target protein responsible for poor sperm quality induced by USP2 deficiency remains unclear, the potential of USP2 as a diagnostic biomarker for male infertility has been highlighted. The level of USP2 expression may be used as a sperm selection marker to detect poor-quality sperm for improving IVF (In Vitro Fertilization) and ICSI (Intracytoplasmic Sperm Injection) outcomes.

### 5.3. USP7

USP7 has been shown to play a crucial role in the regulation of gene expression, DNA repair, and apoptosis [105,106]. In pachytene spermatocytes, SCML2 (a testis-specific polycomb protein) localizes to the XY body, a transcriptionally silenced nuclear region, where it recruits the USP7 [87]. SCML2-dependent recruitment of USP7 to the XY body is critical for regulating H2A ubiquitination [87], a modification linked to transcriptional repression in meiotic cells [88,89]. Mice lacking *Scml2* have increased levels of H2A ubiquitination on the XY body and exhibit spermatogenic failure characterized by increased apoptotic spermatocytes, leading to male infertility. USP7 has been known to regulate cell proliferation and apoptosis by stabilizing p53 [107]. In response to DNA damage, USP7 stabilizes p53 by deubiquitinaton, triggering apoptosis to remove damaged cells [108]. This suggests that dysregulation of USP7 leads to enhanced apoptosis, resulting in a loss of germ cells. Indeed, a significantly increased level of *USP7* expression has been found in infertile men with oligospermia (low sperm count) [109]. This suggests that USP7 may be developed as a potential biomarker for the prognosis, detection, and screening of oligospermia. Furthermore, a recent study has shown that USP7 may play a role in chromatin remodeling during spermiogenesis in mice [110]. USP7 negatively regulates histone ubiquitination (H2A and H2B) through interaction with Fam170a (a stress-inducible actin-binding protein) in spermatids, promoting histone-to-protamine exchange, which is essential for chromatin remodeling for sperm maturation.

### 5.4. USP8/UBPY

USP8 is highly increased in round and elongating spermatids and contributes to the formation of the mouse acrosome, which is indispensable for fertilization [111,112]. During spermiogenesis, monoubiquitination plays a role in the formation of the acrosome (cap-like structure on sperm head) by regulating vesicular transport and protein sorting [113]. Proper acrosome biogenesis is essential for male fertility because the acrosome contains the enzymes necessary for the sperm to penetrate the egg’s protective layers (zona pellucida) during fertilization [114,115]. USP8 interacts with MSJ-1 (male germ cell-specific J-domain protein 1), a sperm-specific molecular chaperone, suggesting that USP8 can regulate the stability and localization of essential proteins required for sperm maturation as a chaperone [111,116]. In addition, USP8, through its MIT (microtubule interacting and trafficking/transport) domain, directly associates with spermatid microtubules, likely linking vesical trafficking and cytoskeleton machinery [112]. Furthermore, USP8 is responsible for sorting and recruiting the MET, a receptor tyrosine kinase, to the developing acrosome, ultimately influencing sperm function for fertilization [117]. Notably, the SNP re7174015 located in the USP8 gene has been identified to be associated with severe impairments in human spermatogenesis, particularly in cases of non-obstructive azoospermia [118,119]. The relationship between this SNP and the function of USP8 is unknown; however, it is known that USP8-rs7174015 is located in the intron of USP8 and can modify the binding sites for transcription factors involved in spermatogenesis, which may affect USP8 expression and lead to male infertility, highlighting the potential of USP8 as a genetic marker for male infertility.

### 5.5. USP9X

USP9X (Ubiquitin-Specific Peptidase 9 X-Linked) is highly expressed in spermatogonia and primary spermatocytes, suggesting its critical role in the early stage of spermatogenesis [120]. *Usp9x* conditional knock-out mice exhibit spermatogenic failure, characterized by increased apoptosis in the early spermatocytes, leading to male infertility [120]; this suggests its crucial role in the transition from mitotic proliferation of spermatogonia to meiotic initiation. Moreover, USP9X variants have been identified in infertile men with oligospermia [121,122]. USP9X has previously been reported to deubiquitinate and stabilize pro-survival MCL1 (Myeloid Cell leukemia 1), thereby promoting cell survival [123]. In addition, USP9X is involved in the regulation of HR repair by stabilizing BRCA1 [124,125], suggesting that USP9X deficiency may increase genome instability, leading to massive germ cell apoptosis and consequently impaired spermatogenesis.

### 5.6. USP9Y

The Y-linked gene *USP9Y* was historically linked to male infertility. *USP9Y* is located on the azoospermia factor region a (*AZFa*) of the Y chromosome, which contains genes critical for spermatogenesis [126,127]. Deletions affecting *USP9Y* have been associated with azoospermia or severe oligospermia [128,129]. However, the view of *USP9Y* as an essential gene for male fertility has changed after finding men with complete *USP9Y* deletions who still have normal fertility [130], suggesting that USP9Y is not strictly required for spermatogenesis, or that compensatory mechanisms may exist. 

### 5.7. USP11

USP11, which encodes X-linked *USP11*, is predominantly expressed in human SSCs [131]. Silencing USP11 leads to decreased proliferation and increased apoptosis in human SSCs [131]. The pro-survival effects of USP11 are mediated by its interaction with HOXC5 (Homeobox C5). USP11 stabilizes HOXC5 by deubiquitination, which then promotes the canonical WNT/β-catenin signaling pathway to maintain SSC proliferation. In addition, lower levels of USP11 expression were found in infertile men with non-obstructive azoospermia [131], suggesting that USP11 dysfunction may contribute to male infertility. Additionally, USP11 has been shown to be involved in DNA damage repair by stabilizing histone H2AX and BRCA2 through deubiquitination [132]. Moreover, USP11 also plays a critical role in regulating cell cycle progression and DNA damage responses by stabilizing p21, a cell cycle inhibitor [133]. However, despite its potential roles in spermatogenesis, *Usp11*-deficient mice are fertile [134]. A possible explanation for this observation could be significant genetic compensation through the upregulation of related USPs in mice.

### 5.8. USP12

USP12 requires interaction with two cofactors, UAF1 (WDR48) and WDR20, for its enzymatic activity [94]. A recent study has shown that USP12 interacts with UAF1 in mouse testes [96], suggesting that it may play a role in spermatogenesis as a complex with UAF1. However, the biological function of USP12 during spermatogenesis has not been studied yet. Given its known functions [135], the possible mechanisms involved in spermatogenesis include the following: (i) Genome maintenance: USP12 has been reported to be associated with DNA damage repair [136,137], suggesting that USP12 can play a role in the genome stability of germ cells. (ii) Regulation of apoptosis: USP12 has been shown to deubiquitinate BAX (BCL2-associated X protein), a proapoptotic protein [138], suggesting that USP12 can affect BAX activity, thus regulating germ cell apoptosis. (iii) Cell cycle regulation: USP12 influences cell proliferation by regulating the transcription of genes like c-Myc [139,140]. The role of USP12 in cell cycle regulation could affect SSC proliferation and maintenance. (iv) Androgen receptor (AR) signaling pathway: USP12, in complex with UAF1 and WDR20, deubiquitinates AR to enhance receptor stability and transcriptional activity [141], which may contribute to maintaining spermatogenesis (v) Deubiquitination of histones: Histone ubiquitination is involved in chromatin remodeling during sperm maturation [88,89]. USP12 is known to deubiquitinate histones H2A and H2B [142], suggesting that USP12 may participate in the regulation of gene expression and chromatin remodeling in germ cells.

### 5.9. USP14

USP14 is important for post-meiotic differentiation. Genetic studies using both Drosophila and mice have shown that a loss of USP14 function leads to male infertility characterized by defects in sperm maturation [143,144]. The absence of Drosophila *Usp14* disrupts normal spermatid differentiation and reduces free ubiquitin levels in testes [143,144]. *Usp14*-deficient mice (also known as ataxia mice) display reduced sperm counts and produce malformed spermatozoa, including two-tailed spermatozoa and decapitated sperm bodies [143], highlighting its role in regulating spermiogenesis. USP14 is associated with the 26S proteasome and exerts a dual role to both activate and inhibit protein degradation [145], suggesting that USP14 possibly coordinates the highly organized process of spermiogenesis through its interaction with the proteasome that is specifically required for sperm maturation.

### 5.10. USP26

USP26 is an X-linked deubiquitinase that plays a critical role in spermatogenesis [146,147], including chromatin remodeling, protein stability, and androgen receptor signaling. USP26 is specifically expressed in the testis throughout all stages and it has been proposed to be involved in histone removal and in the regulation of protein turnover during spermatogenesis [148,149,150]. USP26 has been linked to the regulation of androgen receptor (AR). USP26 binds to AR and removes ubiquitin chains, preventing its degradation and increasing its transcriptional activity, which is essential for the maintenance of normal spermatogenesis [151,152]. Recently, it has been demonstrated that USP26 is localized on the XY body during pachytene, essential for the proper pairing and synapsis of the sex chromosomes [153]. Disruption of *USP26* causes incomplete sex chromosome pairing, resulting in XY aneuploid spermatozoa and male infertility in both humans and mice. Beyond spermatogenesis, it has been shown that USP26 (along with USP37) is recruited to DSBs and regulates HR repair by removing RNF168-induced ubiquitin conjugates on histones (H2A, H2AX), specifically counteracting the BRCA1-RAP80 complex [154], suggesting its potential role in maintaining genome stability in germ cells. USP26 also regulates pluripotency in embryonic stem cells by stabilizing components of polycomb-repressive complex1 (PRC1) which are responsible for histone H2A ubiquitination, thereby repressing transcription of pluripotency genes such as *Sox2* and *Nanog* [155]. This suggests that USP26 might be implicated in the regulation of differentiation and proliferation of SSCs. Mutations or lower expression of *USP26* is associated with severely impaired spermatogenesis in men [150,156,157,158,159]. The genetic variants identified in *USP26* are associated with various forms of male infertility, including Sertoli cell-only syndrome, azoospermia, and asthenoteratozoospermia (abnormal sperm motility and morphology). However, most of the identified *USP26* mutations have no significant change in catalytic activity [160,161]. Therefore, further investigation is needed to clarify the direct association between these USP26 mutations and male infertility. 

### 5.11. USP42

Usp42 expression in testis fluctuates throughout the different stages of spermatogenesis [162]. Usp42 is expressed from 2 weeks after birth and reaches its peak levels at round spermatid stages, then gradually decreases, suggesting that USP42 may have a role during the early and mid-stages of spermatogenesis. USP42 has been shown to deubiquitinate and stabilize p53 in response to cellular stress, inducing p53-dependent gene expression [163]. Given p53’s role in regulating the cell cycle and apoptosis, this interaction could be relevant for controlling the proliferation and survival of germ cells during spermatogenesis. Additionally, USP42 can deubiquitinate histone H2B, regulating transcriptional activity [164], suggesting its potential role in transcriptional regulation during spermatogenesis. USP42 is also involved in homologous recombination repair by promoting BRCA1 loading to DNA double-strand break (DSB) sites [165]. Despite its potential role in spermatogenesis, specific knock-out phenotypes (e.g., infertility or specific spermatogenic arrest) for *Usp42* have not been identified.

### 5.12. USP44

USP44 (Ubiquitin-specific protease 44) is a critical DUB that plays a role in regulating embryonic stem cell differentiation and maintaining pluripotency by controlling the level of histone H2B monoubiquitination. USP44 is a direct target of the pluripotency factor Oct4 [166]. In embryonic stem cells, USP44 is highly expressed but is downregulated during differentiation [167]. USP44 acts as an antagonist to the E3 ligase RNF20, which is responsible for the monoubiquitination of histone H2B [167]. By counteracting RNF20, USP44 maintains low levels of H2B ubiquitination required for stem cell pluripotency. USP44 is highly expressed in testis compared to other tissues [168], suggesting that it plays a significant regulatory role during spermatogenesis. While many studies have confirmed the role of USP44 in embryonic stem cells, specific details about its exact function in SSCs are less defined. However, as a critical regulator of the stem cell state, USP44 may be implicated in the maintenance of undifferentiated states of SSCs. Besides stem cell maintenance, USP44 is known to be involved in regulating cell cycle progression and genomic stability, which may also contribute to normal spermatogenesis. USP44 plays a key role in mitosis and cell proliferation by regulating the mitotic spindle assembly checkpoint, thereby preventing premature anaphase onset and ensuring that chromosomes segregate properly during mitosis [169]. USP44 also contributes to the DNA damage response by promoting efficient DNA repair at DSBs, where it deubiquitinates histone H2A, restricting the chromatin recruitment of DNA repair proteins [170,171]. Therefore, a similar role of USP44 in regulating DSB repair in meiosis as in mitosis could be hypothesized and needs to be further investigated.

### 5.13. CYLD

CYLD (cylindromatosis) [172] has been known to play a critical role in spermatogenesis, primarily by regulating an early wave of germ cell apoptosis to eliminate excess cells [173]. CYLD negatively regulates the NF-κB signaling pathway, a key regulator of cell survival, by removing K63-linked ubiquitin chains from RIP1 (Receptor-interacting Protein 1) which are central to activating NF-κB [174,175]. The loss of *Cyld* leads to the activation of NF-κB and the subsequent expression of anti-apoptotic genes like *Bcl2*. This constitutive activation of NF-κB results in aberrant accumulation of spermatogonia and early spermatocytes due to decreased apoptosis [173]. While early-stage germ cells accumulate, later stages of spermatogenesis are severely impaired, resulting in a lack of spermatids and spermatozoa. CYLD has recently been revealed to interact with microtubules and modulate microtubule dynamics [176,177]. Moreover, the microtubule regulatory activity of CYLD is essential for its functions in cell cycle progression, especially in the regulation of mitotic entry, spindle formation and orientation, and cytokinesis [178,179]. CYLD interacts with PLK1, an important mitotic kinase, and regulates its activity by deubiquitinating K63-linked ubiquitin chains on PLK1 [180]. Mitotic spindle formation is regulated by direct interaction with centrosomal protein CEP192 [181]. Collectively, CYLD dysregulation may have multilevel cellular impacts in spermatogenesis where CYLD is mutated or its expression is lost. Although studies using mouse models demonstrate a critical role of CYLD in spermatogenesis, the direct link between *CYLD* mutations and human male infertility remains less clear.

### 5.14. UCHL1 and UCHL3

Ubiquitin C-terminal hydrolase L1 (UCHL1) is a pivotal member of the UCH family of deubiquitinating enzymes. UCHL1 and UCHL3 are the two most predominant isozymes in UCHs, sharing more than 50% sequence identity; however, their expression pattern and function in germ cells are distinct [182]. UCHL1 is highly expressed in undifferentiated spermatogonia and Sertoli cells [183], while UCHL3 is primarily expressed in meiotic pachytene spermatocytes and post-meiotic spermatids [184,185]. During mitotic division of undifferentiated spermatogonia, the asymmetric segregation of the UCHL1 protein between two daughter cells follows asymmetric division [186]. UCH-L1 expression levels in spermatogonia are associated with SSC self-renewal and differentiation as well as cell proliferation, making it an ideal candidate biomarker for asymmetric distribution during SSCs self-renewal and differentiation. A recent study using *Uchl1*-deficientmice demonstrated that UCHL1 plays a critical role in differentiation competence and metabolic regulation in SSCs [183]. While initial spermatogonia in *Uchl*-deficient mice present comparable to those of wild-type mice, they show a loss of long-term differentiation capacity, leading to progressive degeneration of seminiferous tubules. Furthermore, *Uchl1*-deficient mice-derived SSCs have a reduced capacity to regenerate full spermatogenesis after transplantation in vivo. Interestingly, both UCHL1 deficiency and UCHL1 overexpression impair normal spermatogenesis. *Uchl1*-deficient gad mice exhibit decreased apoptosis during the first round of spermatogenesis, resulting in an increased number of spermatogonia [187]. However, with age, *Uchl1*-deficient mice show progressive degeneration of the seminiferous tubules with a significant reduction in spermatogonia and primary spermatocytes. UCHL1 is also associated with later stages of spermatogenesis [187]. *Uchl1*-deficient gad mice display reduced sperm motility and an increased number of abnormal spermatozoa. Conversely, overexpression of UCHL1 also disrupts spermatogenesis [188]. Transgenic mice overexpressing UCHL1 display meiotic arrest of pachytene spermatocytes and increased apoptosis, resulting in a complete loss of post-meiotic germ cells.

UCHL3 is primarily expressed in pachytene spermatocytes and post-meiotic spermatids in mice [184], particularly in the acrosome and flagella [189]. A recent study has shown that decreased UCHL3 levels and activity are observed in men with asthenozoospermia (reduced sperm motility) and oligoasthenozoospermia (low sperm count and reduced sperm motility) [189]. Moreover, UCHL3 levels and activity are positively correlated with sperm count, motility, and fertilization rate, further supporting the importance of UCHL3 in sperm maturation and suggesting its potential as a biomarker for male infertility. Additionally, UCHL3 has been shown to be involved in DNA damage repair. Phosphorylated UCHL3 deubiquitinates RAD51 following DNA damage, promoting the interaction between RAD51 and BRCA2, which is required for HR repair [190]. UCHL3 also plays a role in facilitating cell viability upon DNA damage by inhibiting KU80 ubiquitination and enhancing KU80 retention at sites of DNA damage [191], suggesting its potential role in the maintenance of genome stability in germ cells, which may contribute to male fertility.

Compared to UCHL1 and UCHL3, the involvement of UCHL4 and UCHL5 in spermatogenesis remains unclear. Although UCHL4 is primarily expressed in spermatogonia and UCHL5 is expressed in spermatocytes and spermatids [184], their specific protein interactions within these cells are not fully defined. Notably, UCHL5 constitutes a component of proteasomes and INO80 chromatin remodeling complexes, where the DUB activity of UCLH5 is activated and inhibited, respectively [192,193,194,195,196]. In addition, a recent report has implicated UCH-L5 and INO80G (INO80 complex subunit G) as key factors of DNA damage repair [197,198], suggesting the potential role of UCHL5 in the maintenance of genome integrity during spermatogenesis. Further research is needed to explore the implication of this interaction in spermatogenesis and male fertility.

### 5.15. OTUD6A

The Ovarian Tumor (OTU) family of deubiquitinases (DUBs) consists of 16 enzymes in humans that exhibit polyubiquitin linkage-specific activity and regulate diverse cellular processes, including immune responses, DNA damage repair, cell cycle regulation, and signaling pathways [199,200]. OTUDs are a key subfamily of the OTU family, with important DUB activities, and have been linked to various human diseases [201]. OTUD6A is highly expressed in the mouse testis and its expression begins in type B spermatogonia during spermatogenesis and continues through the pachytene stage of spermatocytes [202]. *Otud6a*-deficient mice exhibit subfertility characterized by elevated germ cell death and a low sperm count, highlighting its essential role in spermatogenesis [202]. OTUD6A has been shown to interact with and stabilize various oncogenic proteins including c-MYC, BRG1 (chromatin remodeler), and AR (androgen receptor), promoting tumorigenesis [203]. By stabilizing these oncogenic proteins, OTUD6A may influence cell cycle progression, critical for stem cell division during spermatogenesis. OTUD6A may also be required for proper meiotic progression, though its specific targets in the testis have not been identified.

**Table 1:** List of DUBs mentioned in this review and their proposed testicular substrates, function, and phenotype.

DUB	Ub Chain	Substrates	Function	Model	Phenotype	References
***USPs (Ubiquitin specific proteases)***						
USP1	Mono-Ub	FANCD2	DNA damage repair	Mouse	Infertility, loss of germ cells	[93]
				Human	SNP-associated NOA	[50,103]
USP2	K63	Unknown	Sperm motility, Fertilization	Mouse	Subfertility, reduced sperm motility, reduced fertilization	[104]
USP7	Mono-Ub, K48, K63	H2A, H2B, P53?	Chromatin remodeling	Mouse	Infertility, increased apoptosis	[87,110]
				Human	Oligospermia (Increased USP7 expression)	[109]
USP8	Mono-Ub, K48, K63	MET, MSJ-1	Acrosome biogenesis	Human	SNP-associated idiopathic infertility	[119]
USP9X	K48, K33,	MCL1?	SSC differentiation	Mouse	Infertility, loss of germ cells	[120]
				Human	Oligoasthenozoospermia	[121,122]
USP9Y		Unknown	Male germ cell development	Human	Azoospermia, oligospermia	[128,129]
USP11	K48	HOXC5	SSC maintenance	Human	NOA (low USP11 expression)	[131]
USP12	K48	AR	Unknown			[141]
USP14	K48	Unknown	Spermiogenesis	Mouse	Oligospermia, malformed sperm	[143]
USP26	Mono-Ub, K48	H2A, AR	Chromosome pairing, AR signaling	Mouse	Infertility, XY aneuploid sperm	[151,153]
				Human	Polymorphisms associated with OA and NOA	[156,157,158,159]
USP42	Mono-Ub	H2B	Gene expression?			[163,164]
USP44	Mono-Ub	H2B	SSC maintenance?			[167,170]
CYLD	K63	RIP1	Early-wave apoptosis	Mouse	Infertility, defect in early-wave apoptosis, loss of germ cells	[173]
***UCHs (Ubiquitin C-terminal hydrolases)***						
UCHL1		Unknown	SSC differentiation, Sperm maturation	Mouse	Defect in early-wave apoptosis, malformed spermatozoa, reduced sperm motility	[187]
UCHL3	K48	RAD51? KU80?	Sperm maturation	Mouse	Reduced sperm count, sperm motility, and fertility	[189,190,191]
UCHL4		Unknown	Unknown			[184]
UCHL5	K48	Unknown	Unknown			[192,197]
***OTUs (Otubain proteases)***						
OTUD6A	K48	Unknown	Unknown	Mouse	Subfertility, increased apoptosis, oligospermia	[202]

Abbreviations: OA, obstructive azoospermia; NOA, non-obstructive azoospermia; AR, androgen receptor; SSC, spermatogonial stem cell; SNP, single nucleotide polymorphism; A question mark indicates that it is undefined and further studies are required.

## 6. Clinical Significance of DUBs as a Biomarker for Male Infertility

In the mammalian reproductive system, sperm ubiquitination in the epididymis acts as a quality control mechanism, tagging defective, immature, or abnormal spermatozoa for elimination via phagocytosis in the epididymis during maturation [204,205]. This process marks surface proteins on defective sperm for degradation and is associated with poor sperm quality [206]. Studies have demonstrated that elevated levels of ubiquitination on the sperm surface strongly correlate with morphological abnormalities, low sperm concentration, and poor motility [206], making it a potential diagnostic marker. Measuring sperm ubiquitination may be an effective diagnostic tool for male infertility which allows for the separation of high-quality sperm for assisted reproductive technologies (ARTs). However, conflicting evidence regarding a positive correlation between sperm ubiquitination and good sperm quality exists [207], inspiring the development of more efficient diagnostic strategies [208].

Deubiquitinating enzymes (DUBs) are critical regulators of the ubiquitin–proteasome system (UPS) that play a significant role in spermatogenesis, sperm maturation, and fertilization. The assessment of DUB levels and activity in semen may provide insight into male infertility, particularly by identifying dysfunctional sperm. Several DUBs are emerging as key molecular biomarkers for diagnosing male infertility. UCHL3 is one of the most prominent DUB biomarkers. UCHL3 is localized in the acrosome and tail of spermatozoa, and its expression levels and enzymatic activity are positively correlated with sperm count, concentration, and motility [189]. The expression and activity of UCHL3 may serve as a positive indicator of fertilization rates in In Vitro Fertilization (IVF). USP2 plays a role in spermiogenesis and the transition of sperm through the epididymis [104], which is essential for the formation of high-quality sperm, suggesting that USP2 expression may serve as a diagnostic marker for male infertility. USP8 is involved in the regulation of the acrosome reaction [111,112], which enables the penetration of the oocytes and subsequent fertilization. This suggests that USP8 is a promising biomarker for predicting fertilization rates. USP26 is expressed specifically in the testes and is considered a significant marker for male infertility. Polymorphisms in the *USP26* gene have been associated with human male infertility and may be used as indicators of defective spermatogenesis [150,156,157,158,159]. Lastly, the expression of USP7 is significantly elevated in men with oligospermia [109], highlighting the potential of USP7 as a molecular marker for the development of oligospermia.

## 7. Male Infertility and Cancer

A recent study found that male infertility is associated with a higher risk of developing cancer [209,210,211]. This association is likely due to shared biological pathways, including cell cycle regulation and DNA damage repair. Defects in genes involved in these processes might induce both spermatogenic failure and cancer development. Therefore, dysregulation of DUBs involved in DNA repair may be associated with both infertility and cancer. This connection suggests that DUB mutations associated with male infertility can be considered as a biomarker to assess future risk of developing cancer in infertility patients.

DUBs have emerged as promising anticancer targets in recent years [17,27,212,213,214]. The aberrant expression and dysregulation of DUBs are frequently found in many cancers, making them promising therapeutic targets for cancer therapy [215,216]. Small-molecule inhibitors targeting DUBs have been developed and are in preclinical development for various cancers [217,218]. However, the essential role of DUBs in spermatogenesis suggests that DUB inhibitors used for cancer treatment could potentially disrupt normal testicular function and cause infertility. Therefore, targeting DUBs as anticancer therapy requires careful consideration of its potential impact on fertility.

## 8. Conclusions

Spermatogenesis is a complex process crucial for male fertility involving the differentiation of spermatogonial stem cells into mature spermatozoa. Disruption of this highly ordered process is directly linked to male infertility. Therefore, understanding the molecular pathways involved in spermatogenesis is essential for addressing the challenge of male fertility. In this review, we summarized the significant progress in understanding the key roles of DUBs implicated in spermatogenesis. There is accumulating evidence that DUBs play a crucial role during spermatogenesis, and defects in specific DUBs have been associated with male infertility in both mice and humans. The target proteins that DUBs impact during spermatogenesis are still being studied, but identifying these proteins is crucial for understanding their exact molecular function.

In conclusion, deubiquitinating enzymes are essential for normal spermatogenesis, and their dysfunction has been associated with male infertility. Further investigation to identify the molecular mechanism of their regulatory roles in spermatogenesis will pave the way for potential future diagnostic tools and targeted treatments.

## Data Availability

No new data were created or analyzed in this study.
